# Evaluation of Soft Tissue Sarcoma Response to Preoperative Chemoradiotherapy Using Dynamic Contrast-Enhanced Magnetic Resonance Imaging

**DOI:** 10.18383/j.tom.2016.00202

**Published:** 2016-12

**Authors:** Wei Huang, Brooke R. Beckett, Alina Tudorica, Janelle M. Meyer, Aneela Afzal, Yiyi Chen, Atiya Mansoor, James B. Hayden, Yee-Cheen Doung, Arthur Y. Hung, Megan L. Holtorf, Torrie J. Aston, Christopher W. Ryan

**Affiliations:** 1 Advanced Imaging Research Center,; 2 Knight Cancer Institute,; 3 Department of Diagnostic Radiology,; 4 Division of Hematology and Medical Oncology,; 5 Department of Public Health and Preventive Medicine,; 6 Department of Pathology,; 7 Department of Orthopaedics and Rehabilitation, and; 8 Department of Radiation Medicine, Oregon Health & Science University, Portland, Oregon

**Keywords:** soft tissue sarcoma, therapy response, DCE-MRI, pharmacokinetic modeling, Shutter-Speed model

## Abstract

This study aims to assess the utility of quantitative dynamic contrast-enhanced (DCE) magnetic resonance imaging (MRI) parameters in comparison with imaging tumor size for early prediction and evaluation of soft tissue sarcoma response to preoperative chemoradiotherapy. In total, 20 patients with intermediate- to high-grade soft tissue sarcomas received either a phase I trial regimen of sorafenib + chemoradiotherapy (n = 8) or chemoradiotherapy only (n = 12), and underwent DCE-MRI at baseline, after 2 weeks of treatment with sorafenib or after the first chemotherapy cycle, and after therapy completion. MRI tumor size in the longest diameter (LD) was measured according to the RECIST (Response Evaluation Criteria In Solid Tumors) guidelines. Pharmacokinetic analyses of DCE-MRI data were performed using the Shutter-Speed model. After only 2 weeks of treatment with sorafenib or after 1 chemotherapy cycle, K^trans^ (rate constant for plasma/interstitium contrast agent transfer) and its percent change were good early predictors of optimal versus suboptimal pathological response with univariate logistic regression C statistics values of 0.90 and 0.80, respectively, whereas RECIST LD percent change was only a fair predictor (C = 0.72). Post-therapy K^trans^, v_e_ (extravascular and extracellular volume fraction), and k_ep_ (intravasation rate constant), not RECIST LD, were excellent (C > 0.90) markers of therapy response. Several DCE-MRI parameters before, during, and after therapy showed significant (*P* < .05) correlations with percent necrosis of resected tumor specimens. In conclusion, absolute values and percent changes of quantitative DCE-MRI parameters provide better early prediction and evaluation of the pathological response of soft tissue sarcoma to preoperative chemoradiotherapy than the conventional measurement of imaging tumor size change.

## Introduction

Soft tissue sarcomas are a rare, heterogeneous group of malignancies that can be found in nearly any site in the body, most commonly occurring in the extremities. While surgical resection is the mainstay of treatment, large tumors (>5 cm) with high pathological grade have an unacceptably high rate of metastatic recurrence and subsequent death. Although optimal treatment for high-risk sarcomas of the extremities remains undefined, one strategy involves the use of combination preoperative chemotherapy and radiation. The intent of such treatment is to treat micrometastatic disease in hopes of preventing metastatic relapse, to sensitize the tumor to the effects of radiation with chemotherapy to prevent local tumor recurrence, and to potentially downsize the tumor to facilitate surgical resection. The degree of necrosis found in pathological examination of the tumor specimen after surgery has been suggested to correlate with disease control and overall survival (1). Thus, a noninvasive imaging method that is useful for prediction and evaluation of soft tissue sarcoma response to preoperative therapy could be a valuable tool to enable personalized treatment.

Measurement of imaging tumor size change based on the RECIST (Response Evaluation Criteria In Solid Tumors) guideline (2) is commonly used in clinical trials to evaluate solid tumor response to treatment. However, size change in response to therapy is often found to be preceded by changes in underlying tumor functions (3–7), such as perfusion and permeability, cellularity, and metabolism. As a noninvasive imaging method for evaluation of perfusion and permeability, dynamic contrast-enhanced magnetic resonance imaging (DCE-MRI) is increasingly used in research and early-phase clinical trial settings to assess and, importantly, predict tumor response to treatment (3–5).

In soft tissue sarcomas, because the primary tumors are often heterogeneous in composition, including areas of fibrosis, necrosis, and hemorrhage, radiographic tumor size shrinkage from preoperative therapy is uncommon and does not necessarily correlate with the clinical outcome (8). The reports on soft tissue sarcoma staging and preoperative therapeutic monitoring using DCE-MRI are limited in the literature. In most studies, either qualitative description of the curve shape (9–12) or semiquantitative metrics, such as wash-in and wash-out rates (13, 14) or area-under-the-cure (13), were used in the analysis of DCE-MRI signal intensity time-course data. Although these 2 methods are simple and straightforward without the need for sophisticated mathematical modeling, the results are directly related to MRI signal change—not tissue biology, and are generally dependent on data acquisition protocol details and scanner platforms and settings, making it difficult to compare studies across institutions (15–17). Quantitative analysis of the DCE-MRI data by pharmacokinetic modeling allows estimation of imaging biomarkers that are direct measures of tissue biological properties and, in principle, independent of data acquisition details and scanner platform. In this paper, we report our initial results in the evaluation of soft tissue sarcoma response to preoperative chemoradiotherapy using quantitative DCE-MRI. The DCE-MRI data were analyzed using a pharmacokinetic model that takes into account the finite intercompartmental water exchange kinetics (18, 19).

## Materials and Methods

### Patient Cohort and Study Schema

In this institutional review board-approved and HIPAA (Health Insurance Portability and Accountability Act of 1996)-compliant prospective study, 20 patients (male, 15; female, 5; mean age, 49 years; age range, 25–69 years) with histologically confirmed, ≥5 cm, intermediate- to high-grade soft tissue sarcomas of the extremities, in whom preoperative systemic therapy and surgical resection were planned, provided written informed consent to participate in a longitudinal research MRI study that included DCE-MRI. The tumors were located in the thigh (n = 13), knee (n = 3), and calf (n = 4) regions. The exclusion criteria included indwelling metal, severe claustrophobia, pregnancy, or glomerular filtration rate <30/mL/min/1.73 m^2^.

In total, 12 patients were treated with our institutional-standard chemoradiotherapy regimen consisting of ifosfamide and epirubicin (IE) combined with preoperative hypofractionated radiation (20). Each 21-day chemotherapy cycle included intravenous (I.V.) infusion of epirubicin (30 mg/m^2^/day) over 3–5 minutes on days 1–4 (epirubicin was omitted during cycle 2) and ifosfamide (2.5 g/m^2^/day I.V. infusion) over 90 minutes on days 1–4 along with I.V. hydration, mesna, antiemetics, and filgrastim or pegfilgrastim. Chemotherapy was planned for 3 preoperative and 3 postoperative cycles. Surgery was planned for week 9, and chemotherapy was resumed ∼4 weeks after surgery. External beam radiation therapy was initiated concomitantly at the start of cycle 2 of chemotherapy and consisted of 28 Gy administered as 8 fractions of 3.5 Gy each over 10 days. The other 8 patients were treated on a phase I clinical trial that included the addition of sorafenib (200 mg daily, 400 mg daily, or 400 mg twice daily), a vascular endothelial growth factor receptor tyrosine kinase inhibitor, to the same chemoradiotherapy regimen, except that chemotherapy was administered for 3 rather than 4 days (21). Sorafenib administration began 2 weeks before the first chemotherapy cycle. The clinicopathological characteristics of the patients are presented in Table 1.

**Table 1.** Clinicopathological Characteristics of Patients

Patient Number	Age (year)	Gender	Histologic Tumor Subtype	Tumor Grade	Pre-therapy Size (cm)	Chemotherapy Regimen	NP (%)	Pathological Response
1	55	Male	Myxoid Liposarcoma	Inter	13.5	IE + S	95	Optimal
2	60	Female	Myxoid Liposarcoma	Inter	13.1	IE + S	85	Suboptimal
3	62	Female	Myxofibrosarcoma	Inter	20.6	IE	50	Suboptimal
4	38	Male	Pleomorphic/Undifferentiated/Spindle	Inter	22.5	IE + S	95	Optimal
5	58	Male	Myxoid Liposarcoma	Inter	24.6	IE + S	95	Optimal
6	43	Male	Spindle Cell Sarcoma	Inter	6.4	IE + S	30	Suboptimal
7	58	Male	Pleomorphic/Undifferentiated/Spindle	High	7.3	IE + S	99	Optimal
8	53	Male	Synovial Sarcoma	Inter	12.7	IE + S	60	Suboptimal
9	25	Male	Synovial Sarcoma	Inter	10.9	IE + S	80	Suboptimal
10	40	Female	Pleomorphic Liposarcoma	High	15.9	IE	80	Suboptimal
11	53	Male	Pleomorphic/Undifferentiated/Spindle	High	5.0	IE	99	Optimal
12	26	Male	Myxofibrosarcoma	Inter	10.4	IE	99	Optimal
13	64	Male	Pleomorphic/Undifferentiated/Spindle	High	8.6	IE	98	Optimal
14	33	Male	Synovial Sarcoma	High	8.0	IE	70	Suboptimal
15	57	Male	Pleomorphic/Undifferentiated/Spindle	Inter	9.0	IE	99	Optimal
16	34	Male	Myxoid Liposarcoma	Inter	5.6	IE	90	Suboptimal
17	64	Female	Pleomorphic/Undifferentiated/Spindle	High	5.7	IE	98	Optimal
18	69	Male	Pleomorphic/Undifferentiated/Spindle	High	18.8	IE	90	Suboptimal
19	40	Female	Myxofibrosarcoma	Inter	6.6	IE	5	Suboptimal
20	46	Male	Synovial Sarcoma	Inter	12.8	IE	30	Suboptimal

Abbreviations: NP, necrosis percentage; Inter, intermediate; IE, Ifosfamide + Epirubicin; S, Sorafenib.

Pre-therapy tumor size was the longest diameter (LD) measured from post-contrast DCE-MRI images.

The research MRI examinations were performed before treatment (visit 1, V1), after 2 weeks of sorafenib-only treatment in the phase I trial or after the first IE treatment cycle in the standard regimen (V2), and after completion of preoperative therapy but before surgery (V3). Several patients dropped out of the MRI study at V2 and V3 due to various personal or medical reasons, resulting in completed MRI examinations in 16 patients (on the standard regimen, 9; on the sorafenib trial, 7) at V2 and 12 patients (on the standard regimen, 7; on the sorafenib trial, 5) at V3.

### DCE-MRI Data Acquisition

All the research MRI studies were performed using a 3 T Tim Trio system (Siemens, Healthcare, Erlangen, Germany) with the body coil as the radiofrequency (RF) transmitter and a phased-array body matrix coil (combined with a phased-arrayed spine matrix coil) as the RF receiver. Following scout and multisection axial T_2_-weighted MRI with fat suppression to locate the tumor, 3-dimensional sagittal DCE-MRI data acquisition with fat suppression was conducted using an RF-spoiled gradient-echo sequence, covering the spatial extent of the tumor. The acquisition parameters included flip angle = 10°, echo time/repetition time = 1.5/6.0 milliseconds, a parallel imaging acceleration factor of 2, field of view = 24–36 cm, in-plane matrix size = 448 × 224, and section thickness = 5.0 mm. The total acquisition time for a DCE-MRI series was ∼10 minutes for 36–80 frames of image volume of 12–30 sections each, with 6.8–16.0 seconds temporal resolution. The variations in the number of frames, the number of sections per volume, and temporal resolution were because of the differences in the tumor size. The I.V. injection of the contrast agent (CA), gadoteridol [Gd(HP-DO3A), 0.1 mmol/kg at 2 mL/s; ProHance, Bracco Diagnostic Inc.], by a programmable power injector, was timed to commence after acquisition of 5 frames of baseline image volumes, followed by a 20-mL saline flush.

For quantification of the pre-CA T_1_ value, T_10_, proton density-weighted images were acquired immediately before and spatially coregistered with the DCE-MRI scan (22, 23). The data acquisition pulse sequence and parameters were the same as those for the DCE-MRI scan except for flip angle = 5° and repetition time = 50 milliseconds.

### Pharmacokinetic Analysis of DCE-MRI Data

The soft tissue sarcoma region of interest (ROI) was manually drawn by an experienced musculoskeletal radiologist on contiguous post-CA (∼120–180 seconds after CA injection) DCE-MRI image sections that cover the entire spatial extent of the CA-enhanced tumor. The radiologist also measured the LD of the tumor from these images based on the RECIST guidelines (2). Table 1 lists the tumor LD values before treatment (V1).

For each DCE-MRI data set, the voxel signal intensity time-courses within the multisection tumor ROIs were subjected to pharmacokinetic analysis using a 2-compartment 3-parameter fast exchange regime (FXR)-allowed version of the Shutter-Speed model (SSM) (18, 19, 23). The 3 fitting parameters of the FXR-SSM are K^trans^ (rate constant for plasma/interstitium CA transfer), v_e_ (volume fraction of extravascular and extracellular space), and τ_i_ (mean intracellular water molecule lifetime). The τ_i_ parameter is used to account for the finite cross-cell membrane water exchange kinetics. The CA intravasation rate constant, k_ep_, was calculated as k_ep_ = K^trans^/v_e_. Mathematical formulations for the FXR-SSM are described in detail in studies by Yankeelov TE et al., Li X et al., and Tudorica A et al. (18, 19, 23).

Used for pharmacokinetic data analysis, the voxel T_10_ values were determined by comparing signal intensities between the spatially registered proton density-weighted images and the averaged baseline images from the DCE series (22, 23). The arterial input function, the plasma CA concentration time-course, was determined for each individual DCE-MRI data set through direct measurement. An ellipsoidal ROI was placed within the clearly visible femoral artery on a post-CA DCE image section that was approximately through the center of the artery. The ROI signal intensity time-course was recorded and then converted to blood R_1_ (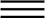
1/T_1_) time-course using the steady-state signal intensity equation for RF-spoiled gradient-echo sequence (22), which was further converted to plasma CA concentration time-course using a linear relationship between R_1_ and CA concentration with a CA relaxivity of 3.8 mM^−1^s^−1^ at 3 T, a fixed pre-CA blood R_1_ of 0.61 s^−1^ (24), and a hematocrit value set at 0.45 (23, 25).

Following the FXR-SSM fittings of the DCE-MRI data, voxel-based multisection parametric maps of the derived pharmacokinetic parameters were generated. The mean pharmacokinetic parameter value of the whole tumor was calculated by averaging the returned voxel parameter values. For each imaging metric, including pharmacokinetic parameters and RECIST LD, the percent changes for later MRI visits relative to V1, V21% (V2 relative to V1) and V31%, were calculated.

### Pathological Analysis

Pathological analysis of the post-therapy resection specimens of each soft tissue sarcoma was performed under light microscopy using standard pathological procedures. The pathologist estimated the amount of viable tumor and the percentage of necrosis. Pathological response to preoperative chemoradiotherapy was classified as either optimal (≥95% necrosis) or suboptimal (<95% necrosis).

### Statistical Analysis

Descriptive statistical analysis was conducted to summarize the pharmacokinetic parameter and RECIST LD values at each MRI visit, as well as the percent changes of these imaging metrics relative to baseline (V1). In assessing the abilities of MRI metrics (absolute values and percent changes) for evaluation of therapy response, the univariate logistic regression (ULR) analysis was used to correlate V1, V2, and V3 MRI metrics, and the corresponding V21% and V31% changes, with dichotomous pathological response endpoints, optimal versus suboptimal. A ULR C statistics value, equivalent to the area under the receiver operating characteristic curve, in the range of 0.9–1.0, indicates an excellent marker; 0.8–0.9, a good marker; 0.7–0.8, a fair marker; and <0.7, a poor marker. Two sample *t* test was used to evaluate the differences in imaging metrics and the corresponding percent changes between the 2 response groups, as well as between the 2 cohorts that received standard chemoradiotherapy and sorafenib + standard chemoradiotherapy, respectively. The Fisher exact test was used to determine whether there was association between therapy regimen (with and without sorafenib) and response status (optimal versus suboptimal). Pearson correlation analysis was used to examine relationships between MRI metrics and necrosis percentage (NP) of the resection specimens.

## Results

As shown in Table 1, the pathological analyses of the surgical specimens revealed that 9 patients (45%) (5 on the standard regimen and 4 on the sorafenib trial) achieved optimal response to preoperative chemoradiotherapy, while the other 11 patients (7 on the standard regimen and 4 on the sorafenib trial) showed suboptimal response. There was no statistically significant (Fisher exact test, *P* = 1.0) association between the use of sorafenib and pathological response status or any significant (two sample *t* test, *P* > .2) differences in any MRI metric (RECIST LD and pharmacokinetic parameters) at any visit and in the corresponding percent changes between the 2 cohorts on different therapy regimens. Therefore, we combined the 2 patient cohorts in assessing the utility of quantitative DCE-MRI for evaluation of response to preoperative therapy.

Table 2 lists the mean ± standard deviation whole-tumor MRI metric values of the optimal and suboptimal response groups and the corresponding ULR C statistics values for discrimination of the 2 response groups. Only the absolute pharmacokinetic parameters and the V21% and V31% changes with C ≥ 0.7, representing fair or better imaging biomarkers, are listed. The C value (0.69) for V31% RECIST LD change is presented for the purpose of comparison. V1, V2, and V21% metrics were obtained before and 2–3 weeks after the start of therapy, and thus, are potential early predictors of therapy response. The V2 K^trans^ parameter was an excellent (C = 0.9) early discriminator of optimal versus suboptimal pathological response, while V1 and V2 k_ep_, V1 and V21% K^trans^, V21% v_e_, and V21% τ_i_ were fair-to-good (0.7 ≤ C ≤ 0.8) markers for early prediction of response. Compared with good-to-excellent predictive abilities of the K^trans^ and k_ep_ metrics, the V21% change in RECIST LD was just a fair early predictor of response. Several pharmacokinetic metrics obtained after the completion of chemoradiotherapy, including V3 k_ep_, K^trans^ and v_e_, and V31% v_e_, were good-to-excellent (0.8 < C < 1.0) discriminators of optimal versus suboptimal response, whereas the V31% change in RECIST LD was a poor (C < 0.7) marker of response. For the imaging metrics listed in Table 2 with ULR C values ≤0.77, including V21% RECIST LD, the differences between the 2 response groups were not statistically significant (*P* > .05).

**Table 2.** Evaluation of Pathological Response (Optimal vs Suboptimal Response)

MRI Metric	OptimalMean ± SD	SuboptimalMean ± SD	*P* Value	ULR C Value
V3 k_ep_ (min^−1^)	0.11 ± 0.03	0.45 ± 0.40	0.0024	0.97
V3 K^trans^ (min^−1^)	0.02 ± 0.01	0.21 ± 0.26	0.0012	0.94
V3 v_e_	0.15 ± 0.08	0.36 ± 0.24	0.021	0.91
**V2 K^trans^ (min^−1^)**	**0.05 ± 0.03**	**0.20 ± 0.13**	**0.0020**	**0.90**
V31% v_e_	−52% ± 28%	53% ± 92%	0.021	0.84
**V21% K^trans^**	**−38% ± 25%**	**−9% ± 33%**	**0.038**	**0.80**
**V1 k_ep_ (min^−1^)**	**0.32 ± 0.24**	**0.75 ± 0.43**	**0.010**	**0.80**
**V2 k_ep_ (min^−1^)**	**0.29 ± 0.26**	**0.68 ± 0.48**	**0.045**	**0.78**
V31% K^trans^	−68% ± 21%	2% ± 75%	0.043	0.78
V3 τ_i_ (s)	1.42 ± 0.83	0.85 ± 0.84	0.25	0.77
**V1 K^trans^ (min^−1^)**	**0.10 ± 0.09**	**0.21 ± 0.16**	**0.055**	**0.72**
**V21% RECIST LD**	**7% ± 10%**	**−3% ± 8%**	**0.13**	**0.72**
**V21% τ_i_**	**38% ± 66%**	**13% ± 43%**	**0.13**	**0.71**
**V21% v_e_**	**−18% ± 42%**	**15% ± 50%**	**0.16**	**0.70**
V31% RECIST LD	−11% ± 22%	−7% ± 8%	0.66	0.69

Abbreviations: ULR, univariate logistic regression; SD, standard deviation.

*P* value, two sample *t* test; V1, V2, and V21% metrics are bolded as early predictors of therapy response.

**Figure 1.**
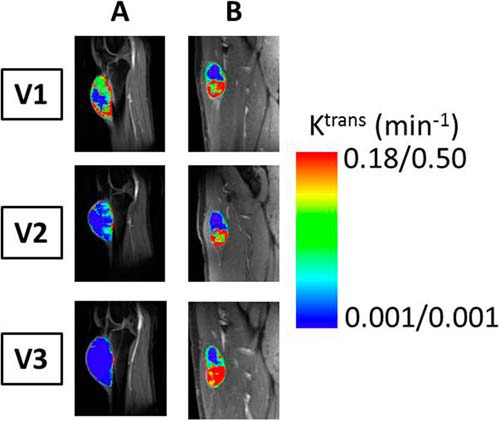
Visit 1 (V1, before therapy), V2 (after 2 weeks of sorafenib administration or 1 chemotherapy cycle), and V3 (after completion of chemoradiotherapy) color parametric K^trans^ maps of 2 soft tissue sarcomas: an optimal (A, left column, 98% necrosis in resection specimen) and a suboptimal (B, right column, 30% necrosis in resection specimen) responder to preoperative therapy. The maps were generated for tumor regions of interest (ROIs) defined on multiple contiguous image sections, and the ones on the image sections through the central portion of the tumors are displayed here. For each tumor, the K^trans^ color scale is kept the same for all 3 visits for easy visualization of therapy-induced changes. The left and right color scales correspond to K^trans^ maps in A and B, respectively.

Figure 1 shows the examples of V1–V3 colored tumor K^trans^ maps from 2 patients with soft tissue sarcoma who had optimal (Figure 1A, left column; Patient 13 in Table 1) and suboptimal (Figure 1B, right column; Patient 6 in Table 1) responses, respectively. The K^trans^ color scales are different for the 2 patients, but it is kept the same throughout the 3 visits for each patient to show changes in the longitudinal study. The 6 panels in Figure 1 are cropped images (without zooming) of K^trans^ maps overlaid on post-CA DCE-MRI image sections that were approximately through the center of the tumor. The field of view of DCE-MRI acquisition was kept the same for all 3 visits for each patient. Thus, it is rather apparent in Figure 1 that there was not only minimal change in the imaging tumor size for each patient but also little difference in tumor size change between the optimal and suboptimal responders in the longitudinal study. However, a substantial decrease in tumor K^trans^ was observed at V2 compared with that at V1, and continued to V3 for the optimal responder, while there were no noticeable K^trans^ changes from V1 to V2, and to V3 for the suboptimal responder.

**Figure 2.**
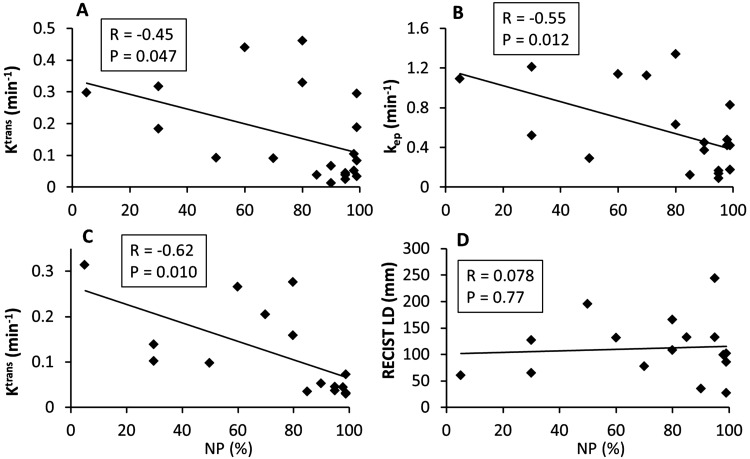
Scatter plots of pathologically measured necrosis percentage (NP) of the resection specimen against K^trans^ (A) and k_ep_ (B) before therapy (V1) and K^trans^ (C) and RECIST LD (D) after 2 weeks of sorafenib administration or 1 chemotherapy cycle (V2). The straight line in each panel represents a linear regression. The Pearson correlation coefficient, R, and *P* values for the 4 imaging metrics are listed in Table 3 and shown in each panel. The data points are from the initial cohort of 20 patients for the V1 metrics (A and B) and the 16 patients who continued to have the V2 MRI studies (C and D).

**Figure 3.**
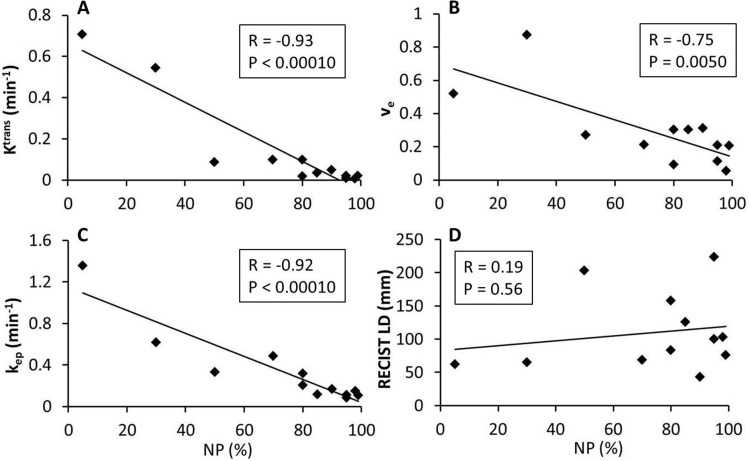
Scatter plots of pathologically measured NP of the resection specimen against MRI metrics after therapy (V3): K^trans^ (A), v_e_ (B), k_ep_ (C), and RECIST LD (D). The straight line in each panel represents a linear regression. The Pearson correlation coefficient, R, and *P* values for the 4 imaging metrics are listed in Table 3 and shown in each panel. The data points are from 12 patients who completed the V3 MRI studies among the initial cohort of 20 patients.

The Pearson correlation coefficient, R, and the *P* values for statistical significance are summarized in Table 3 for correlations between the absolute MRI metric values (and percent changes) and the pathologically measured NP values of the resection specimens. Only the imaging metrics with statistically significant (*P* < .05) correlations with NP are listed, except for V1, V2, V3, V21%, and V31% RECIST LD metrics that are listed for comparison. Figures 2 and 3 show examples of linear regressions between NP and MRI metrics before therapy (Figure 2), at the early stage of therapy (Figure 2), and after therapy (Figure 3). While the negative correlations of V1 K^trans^ (Figure 2A), k_ep_ (Figure 2B), V2 K^trans^ (Figure 2C), V3 K^trans^ (Figure 3A), v_e_ (Figure 3B), and k_ep_ (Figure 3C) with NP were statistically significant (*P* < .05), there were no significant (*P* > .2) associations between any RECIST LD measures and NP.

**Table 3.** Pearson Correlation of MRI Metric with NP

MRI Metric	R	*P*
V3 K^trans^	−0.93	<0.0001
V3 k_ep_	−0.92	<0.0001
V31% K^trans^	−0.89	0.0001
V3 v_e_	−0.75	0.005
V2 K^trans^	−0.62	0.010
V1 k_ep_	−0.55	0.012
V31% v_e_	−0.63	0.028
V1 K^trans^	−0.45	0.047
V21% RECIST LD	0.31	0.25
V31% RECIST LD	−0.20	0.52
V3 RECIST LD	0.19	0.56
V1 RECIST LD	0.071	0.76
V2 RECIST LD	0.078	0.77

Abbreviations: NP, necrosis percentage of the resection specimen; R, Pearson correlation coefficient.

*P* < 0.05 indicates statistically significant correlation.

## Discussion

Consistent with our previously reported findings from the 8 patients with soft tissue sarcoma who were enrolled in the phase I trial of sorafenib + standard chemoradiotherapy (21), this study of a larger cohort of 20 patients shows that changes in tumor functions as measured by quantitative DCE-MRI are superior to changes in RECIST-based imaging tumor size measurement for early prediction of pathological response of soft tissue sarcoma to preoperative therapy, suggesting that therapy-induced tumor functional changes precede changes in tumor size. As an example shown in Table 2, the differences in RECIST LD percent changes between the optimal and suboptimal response groups were much smaller than those in K^trans^ percent changes at the early stage (V21%) of the treatment. Under the condition of 100% sensitivity for early prediction of optimal response (ie, correctly classify all optimal responders in the study cohort), the specificities are 40% and 60% for V21% RECIST LD and V21% K^trans^, respectively, indicating less misclassification of suboptimal responders as optimal responders when V21% K^trans^, instead of V21% RECIST LD, is used as the imaging metric for early prediction of response. In addition to V21% K^trans^, the V1 k_ep_ and V2 K^trans^ parameters were also good-to-excellent (ULR C value ≥0.8) early predictors of optimal versus suboptimal response, and these were significantly associated with NP of the resection specimens in a negative relationship (Table 3 and Figure 2). These results suggest that soft tissue sarcomas with low perfusion and permeability at baseline (before therapy) and/or after 1 chemotherapy cycle may have less angiogenesis-induced abnormal vasculature, and therefore better drug delivery and response. The potential of noninvasive functional imaging methods, such as DCE-MRI, for accurate early prediction of therapy response may have a profound importance in the emerging era of precision and personalized medicine. Early identification of poor responders to a therapy regimen may allow rapid adjustment in treatment planning and spare these patients from ineffective therapies and the associated toxicities.

Our observation of substantial decreases in K^trans^ of the optimally responding soft tissue sarcomas early in the therapy course and after therapy is also in agreement with 2 quantitative DCE-MRI studies of animal soft tissue sarcoma model response to isolated limb perfusion of chemotherapy agents (26, 27). Both reported significant decreases in tumor K^trans^ or K^trans^ variant within hours of chemotherapy administration. Diffusion-weighted MRI (DW-MRI) is another functional imaging method often used to evaluate tumor response to treatment by measuring changes in cellularity (3, 6, 7). In a DW-MRI study (28) to assess soft tissue sarcoma response to chemotherapy, Dudeck et al. showed that significant changes in apparent diffusion coefficient after therapy were detected only when there were substantial changes in tumor volume. Because the changes in tumor size were minuscule (Table 2) throughout the therapy course in this study population, it could be assumed that DW-MRI may not be as effective an imaging tool as DCE-MRI for the evaluation of soft tissue sarcoma response to preoperative chemoradiotherapy.

The post-therapy (V3) K^trans^, k_ep_, and v_e_ parameters all showed strong negative correlations with NP of the resection specimens and were excellent markers (ULR C value >0.9) for discrimination of optimal and suboptimal responders. However, there was no significant correlation between V3 RECIST LD and NP. This suggests that a functional imaging study such as DCE-MRI following preoperative therapy may yield additional information that is potentially useful for surgical planning and subsequent management. The negative correlations of post-therapy K^trans^ and k_ep_ with NP are expected, as increased tumor necrosis is usually associated with decreased perfusion, and thus the DCE-MRI measures of microvascular properties. The similar relationship observed between post-therapy v_e_ and NP is, however, intriguing. With cancer cell death and increased necrosis after the preoperative chemoradiotherapy, the v_e_ value is generally expected to increase with increased necrosis. The opposite was seen in this study and the probable reason for this is that, although defined as extravascular and extracellular volume fraction, v_e_, as measured by DCE-MRI, is, in principle, the putative CA distribution volume fraction. With increased necrosis and decreased viable perfused tumor area, the CA distribution volume fraction, which was reported as an averaged value over the whole tumor volume, also presumably decreased. It is possible that the estimated v_e_ value may actually increase with increased necrosis if the DCE-MRI acquisition time is long enough to allow substantial diffusion of CA molecules into the necrotic area.

The τ_i_ parameter is unique to the SSM method. A recent nuclear magnetic resonance spectroscopy study of yeast cell suspension (29) shows that the reciprocal of τ_i_, k_io_ (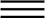
1/τ_i_), the first-order rate constant for equilibrium cellular water efflux, is positively associated with cellular adenosine triphosphate (ATP) levels. The in vivo association of cellular ATP decrease with k_io_ decrease was shown by a DCE-MRI and ^31^P magnetic resonance spectroscopy study of a murine melanoma model treated with lonidamine (30). A review of several studies on cell suspensions, perfused tissue, in vivo animal models, and human breast cancer data implies that k_io_ measures the homeostatic turnover of the cell membrane Na^+^,K^+^-ATPase (31), suggesting that k_io_, as measured with the SSM DCE-MRI method, may be an imaging biomarker of metabolic activity. In this study, both V21% change and V3 tumor τ_i_ were fair markers (Table 2) for discriminating optimal and suboptimal response. The optimal responders had larger percent increase in τ_i_ (or decrease in k_io_) after 2 weeks of sorafenib or after 1 chemotherapy cycle and larger post-therapy τ_i_ values (or smaller k_io_ values) than the suboptimal responders, consistent with presumably greater reduction in metabolic activity in optimal compared with suboptimal responders.

It is interesting to note that even though sorafenib is an antiangiogenic agent (32), there were no significant differences in V21% and V31% decreases of K^trans^ and k_ep_ between the cohort that received sorafenib + standard chemoradiotherapy and the other cohort that received chemoradiotherapy only. IE are cytotoxic chemotherapy agents and not known to be antiangiogenic. The changes in microvascular properties as manifested in K^trans^ and k_ep_ decreases are likely due to the secondary effects of these 2 drugs. It has been suggested (33) that cytotoxic chemotherapy agents may affect the tumor vasculature by interfering with endothelial cell function without causing endothelial cell death or interfering with a specific portion of the angiogenic cascade. This may be the reason why we observed considerable K^trans^ and k_ep_ decreases in tumors treated with IE only. The fact that only small changes were observed in RECIST LD, which is a measurement based on contrast enhancement and thus a measure of well-perfused and permeable (to CA molecules) tumor area, supports the hypothesis that the chemotherapeutics affect tumor vasculature without causing significant endothelial cell death (33). Similarities in V21% and V31% K^trans^ and k_ep_ decreases between the 2 cohorts suggest that the added antiangiogenic effects of sorafenib (to IE treatment) on perfusion and permeability, if any, were not measurable by DCE-MRI in this study.

This study has several limitations. First, the sample size of the study cohort is small. For a total of 20 patients with soft tissue sarcoma, 2 subcohorts were treated with and without sorafenib, respectively. The observation of no significant effects of sorafenib on treatment outcome and changes in DCE-MRI parameters could be because of the small sample size. Thus, it is important to validate the initial findings from this study with a larger patient population in the future. Second, the withdrawal of several patients from the MRI study at V2 and V3 further reduced the sample size in examining the treatment effects and impaired the power of statistical analysis. Again, this limitation can be overcome in the future with a larger study cohort. Third, the mean tumor DCE-MRI parameter values were used in this study to assess soft tissue sarcoma response to preoperative therapy. The tumor heterogeneity that is reflected in the imaging metrics, for example, in the K^trans^ maps shown in Figure 1, was not captured in computing the mean DCE-MRI parameter values. Several recent studies have shown that texture analysis of tumor heterogeneity in parametric maps of kinetic features (34, 35) can be useful for evaluation of breast cancer therapy response. The potential integration of mean values and texture features of DCE-MRI metrics may further improve the effectiveness of quantitative DCE-MRI for assessment of therapy response.

In conclusion, we have shown the utility of quantitative DCE-MRI for early prediction and evaluation of soft tissue sarcoma response to preoperative chemoradiotherapy in this preliminary study of 20 patients with lower extremity tumors. Tumor functional changes as measured by quantitative DCE-MRI parameters such as K^trans^ and k_ep_ provided better early prediction of pathological response outcome than the conventional approach of measuring changes in imaging tumor size. Post-therapy DCE-MRI parameters, not the RECIST LD metric, were found to significantly correlate with percent necrosis of the resection specimens. The SSM-unique τ_i_ parameter could be a useful imaging biomarker of metabolic activity that can be used to evaluate tumor response to therapy.

## References

[1] Eilber FC, Rosen G, Eckardt J, Forscher C, Nelson SD, Selch M, Dorey F, Eilber FR Treatment-induced pathologic necrosis: a predictor of local recurrence and survival in patients receiving neoadjuvant therapy for high-grade extremity soft tissue sarcomas. J Clin Oncol. 2001;19(13):3203–3209. 11432887 10.1200/JCO.2001.19.13.3203

[2] Therasse P, Arbuck SG, Eisenhauer EA, Wanders J, Kaplan RS, Rubinstein L, Verweij J, Van Glabbeke M, van Oosterom AT, Christian MC, Gwyther SG New guidelines to evaluate the response to treatment in solid tumors. European Organization for Research and Treatment of Cancer, National Cancer Institute of the United States, Nation Cancer Institute of Canada. J Natl Cancer Inst. 2000;92(3):205–216. 10655437 10.1093/jnci/92.3.205

[3] Yankeelov TE, Mankoff DA, Schwartz LH, Lieberman FS, Buatti JM, Mountz JM, Erickson BJ, Fennessy FMM, Huang W, Kalpathy-Cramer J, Wahl RL, Linden HM, Kinahan PE, Zhao B, Hylton NM, Gillies RJ, Clarke L, Nordstrom R, Rubin DL Quantitative imaging in cancer clinical trials. Clin Cancer Res. 2016;22(2):284–290. 26773162 10.1158/1078-0432.CCR-14-3336 PMC4717912

[4] O'Connor JPB, Jackson A, Parker GJM, Roberts C, Jayson GC Dynamic contrast-enhanced MRI in clinical trials of antivascular therapies. Nat Rev Clin Oncol. 2012;9(3):167–177. 22330689 10.1038/nrclinonc.2012.2

[5] Leach MO, Morgan B, Tofts PS, Buckley DL, Huang W, Horsfield MA, Chenevert TL, Collins DJ, Jackson A, Lomas D, Whitcher B, Clarke L, Plummer R, Judson I, Jones R, Alonzi R, Brunner T, Koh DM, Murphy P, Waterton JC, Parker G, Graves MJ, Scheenen T, Redpath T, Orton M, Karczmar G, Huisman H, Barentsz J, Padhani A; Experimental Cancer Medicine Centres Imaging Network Steering Committee. Imaging vascular function for early stage clinical trials using dynamic contrast-enhanced magnetic resonance imaging. Eur Radiol. 2012;22(7):1451–1464. 22562143 10.1007/s00330-012-2446-x

[6] Padhani AR, Miles KA Multiparametric imaging of tumor response to therapy. Radiology. 2010;256(2):348–364. 20656830 10.1148/radiol.10091760

[7] Harry VN, Semple SI, Parkin DE, Gilbert FJ Use of new imaging techniques to predict tumor response to therapy. Lancet Oncol. 2010;11(1):92–102. 20129132 10.1016/S1470-2045(09)70190-1

[8] DeLaney TF, Spiro IJ, Suit HD, Gebhardt MC, Hornicek FJ, Mankin HJ, Rosenberg AL, Rosenthal DI, Miryousefi F, Ancukiewicz M, Harmon DC Neoadjuvant chemotherapy and radiotherapy for large extremity soft tissue sarcomas. Int J Radiat Oncol Biol Phys. 2003;56(4):1117–1127. 12829150 10.1016/s0360-3016(03)00186-x

[9] Shapeero LG, Vanel D, Verstraete KL, Bloem JL Fast magnetic resonance imaging with contrast for soft tissue sarcoma viability. Clin Orthop Relat Res. 2002;397:212–227. 10.1097/00003086-200204000-00026 11953613

[10] van Rijswijk CS, Geirnaerdt MJ, Hogendoorn PC, Peterse JL, van Coevorden F, Taminiau AH, Tollenaar RA, Kroon BB, Bloem JL Dynamic contrast-enhanced MR imaging in monitoring response to isolated limb perfusion in high-grade soft tissue sarcoma: initial results. Eur Radiol. 2003;13(8):1849–1858. 12942285 10.1007/s00330-002-1785-4

[11] Del Grande F, Subhawong T, Weber K, Aro M, Mugera C, Fayad LM Detection of soft-tissue sarcoma recurrence: added value of functional MR imaging techniques at 3.0T. Radiology. 2014;271(2):499–511. 24495264 10.1148/radiol.13130844

[12] Soldatos T, Ahlawat S, Montgomery E, Chalian M, Jacobs MA, Fayad LM Multiparametric assessment of treatment response in high-grade soft-tissue sarcomas with anatomic and functional MR imaging sequences. Radiology. 2016;278(3):831–840. 26390048 10.1148/radiol.2015142463 PMC4770945

[13] Viglianti BL, Lora-Michiels M, Poulson JM, Lan L, Yu D, Sanders L, Craciunescu O, Vujaskovic Z, Thrall DE, MacFall J, Charles CH, Wong T, Dewhirst MW Dynamic contrast-enhanced magnetic resonance imaging as a predictor of clinical outcome in canine spontaneous soft tissue sarcomas treated with thermoradiotherapy. Clin Cancer Res. 2009;15(15):4993–5001. 19622579 10.1158/1078-0432.CCR-08-2222 PMC2763531

[14] Noebauer-Huhmann IM, Amann G, Krssak M, Panotopoulos J, Szomolanyi P, Weber M, Czerny C, Breitenseher M, Grabner G, Bogner W, Nemec S, Dominkus M, Funovics P, Windhager R, Trattnig S Use of diagnostic dynamic contrast-enhanced (DCE)-MRI for targeting of soft tissue tumor biopsies at 3T: preliminary results. Eur Radiol. 2015;25(7):2041–2048. 25577522 10.1007/s00330-014-3576-0

[15] Jansen SA, Shimauchi A, Zak L, Fan X, Wood AM, Karczmar GS, Newstead GM Kinetic curve of malignant lesions are not consistent across MRI systems: need for improved standardization of breast dynamic contrast-enhanced MRI acquisition. AJR Am J Roentgen. 2009;193(3):832–839. 10.2214/AJR.08.2025 PMC2938789 19696299

[16] Khalifa F, Soliman A, El-Baz A, El-Ghar MA, El-Diasty T, Gimel'farb G, Ouseph R, Dwyer AC Models and methods for analyzing DCE-MRI: a review. Med Phys. 2014;41(12):124301. 25471985 10.1118/1.4898202

[17] Freed M Effect of protocol parameters on contrast agent washout curve separability in breast dynamic contrast enhanced MRI: a simulation study. Magn Reson Med. 2012;68(2):516–522. 22144368 10.1002/mrm.23234

[18] Yankeelov TE, Rooney WD, Li X, Springer CS Variation of the relaxographic “Shutter-Speed” for transcytolemmal water exchange affects the CR bolus-tracking curve shape. Magn Reson Med. 2003;50(6):1151–1169. 14648563 10.1002/mrm.10624

[19] Li X, Rooney WD, Springer CS A unified pharmacokinetic theory for intravascular and extracellular contrast agents. Magn Reson Med. 2005;54(6):1351–1359. [Erratum in Magn Reson Med 2006;55(5):1217]. 16247739 10.1002/mrm.20684

[20] Ryan CW, Montag AG, Hosenpud JR, Samuels B, Hayden JB, Hung AY, Mansoor A, Peabody TD, Mundt AJ, Undevia S Histologic response of dose-intense chemotherapy with preoperative hypofractionated radiotherapy for patients with high-risk soft tissue sarcomas. Cancer. 2008;112(11):2432–2439. 18348295 10.1002/cncr.23478

[21] Meyer JM, Perlewitz KS, Hayden JB, Doung YC, Hung AY, Vetto JT, Pommier RF, Mansoor A, Beckett BR, Tudorica A, Mori M, Holtorf ML, Afzal A, Woodward WJ, Rodler ET, Jones RL, Huang W, Ryan CW Phase I trial of preoperative chemoradiation plus Sorafenib for high risk extremity soft tissue sarcoma with dynamic contrast-enhanced magnetic resonance imaging correlates. Clin Cancer Res. 2013;19(24):6902–6911. 24132922 10.1158/1078-0432.CCR-13-1594 PMC3869565

[22] Huang W, Wang Y, Panicek DM, Schwartz LH, Koutcher JA Feasibility of using limited-population-based average R_10_ for pharmacokinetic modeling of osteosarcoma dynamic contrast-enhanced MRI data. Magn Reson Imaging. 2009;27(6):852–858. 19282123 10.1016/j.mri.2009.01.020 PMC2722921

[23] Tudorica A, OH KY, Chui SYC, Roy N, Troxell ML, Naik A, Kemmer K, Chen Y, Holtorf ML, Afzal A, Springer CS, Li X, Huang W Early prediction and evaluation of breast cancer response to neoadjuvant chemotherapy using quantitative DCE-MRI. Transl Oncol. 2016;9(1):8–17. 26947876 10.1016/j.tranon.2015.11.016 PMC4800060

[24] Lu H, Clingman C, Golay X, van Zijl PC Determining the longitudinal relaxation time (T1) of blood at 3.0 Tesla. Magn Reson Med. 2004;52(3):679–682. 15334591 10.1002/mrm.20178

[25] Huang W, Chen Y, Fedorov A, Li X, Jajamovich GH, Malyarenko DI, Aryal MP, LaViolette PS, Oborski MJ, O'Sullivan F, Abramson RG, Jafari-Khouzani K, Afzal A, Tudorica A, Moloney B, Gupta SN, Besa C, Kalpathy-Cramer J, Mountz JM, Laymon CM, Muzi M, Kinahan PE, Schmainda K, Cao Y, Chenevert TL, Taouli B, Yankeelov TE, Fennessy FMM, Li X The impact of arterial input function determination variations on prostate dynamic contrast-enhanced magnetic resonance imaging pharmacokinetic modeling: a multicenter data analysis challenge. Tomography. 2016;2(1):56–66. 27200418 10.18383/j.tom.2015.00184 PMC4869732

[26] Preda A, Wielopolski PA, ten Hagen TLM, van Vliet M, Veenland JF, Ambagtsheer G, van Tiel ST, Vogel MW, Eggermont AMM, Krestin GP, van Dijke CF Dynamic contrast-enhanced MRI using macromolecular contrast media for monitoring the response to isolated limb perfusion in experimental soft-tissue sarcomas. MAGMA. 2004;17(3–6):296–302. 15480945 10.1007/s10334-004-0050-z

[27] Alic L, van Vliet M, Wielopolski PA, ten Hagen TL, van Dijke CF, Niessen WJ, Veenland JF Regional heterogeneity changes in DCE-MRI as response to isolated limb perfusion in experimental soft-tissue sarcomas. Contrast Media Mol Imaging. 2013;8(4):340–349. 23613437 10.1002/cmmi.1528

[28] Dudeck O, Zeile M, Pink D, Pech M, Tunn PU, Reichardt P, Ludwig WD, Hamm B Diffusion-weighted magnetic resonance imaging allows monitoring of anticancer treatment effects in patients with soft-tissue sarcomas. J Magn Reson Imaging. 2008;27(5):1109–1113. 18425832 10.1002/jmri.21358

[29] Zhang Y, Poirier-Quinot M, Springer CS, Balschi JA Active trans-plasma membrane water cycling in yeast is revealed by NMR. Biophys J. 2011;101(11):2833–2842. 22261073 10.1016/j.bpj.2011.10.035 PMC3297792

[30] Nath K, Paudyal R, Nelson DS, Pickup S, Zhou R, Leeper DB, Heitjan DF, Springer CS, Poptani H, Glickson JD Acute changes in cellular-interstitial water exchange rate in DB-1 melanoma xenografts after lonidamine administration as a marker of tumor energetics and ion transport. Proc Intl Soc Magn Reson Med. 2014;22:2757.

[31] Springer CS Jr., Li X, Tudorica LA, OH KY, Roy N, Chui SYC, Naik AM, Holtorf ML, Afzal A, Rooney WD, Huang W Intratumor mapping of intracellular water lifetime: metabolic images of breast cancer? NMR Biomed. 2014;27(7):760–773. 24798066 10.1002/nbm.3111 PMC4174415

[32] Wilhelm SM, Carter C, Tang L, Wilkie D, McNabola A, Rong H, Chen C, Zhang X, Vincent P, McHugh M, Cao Y, Shujath J, Gawlak S, Eveleigh D, Rowley B, Liu L, Adnane L, Lynch M, Auclair D, Taylor I, Gedrich R, Voznesensky A, Riedl B, Post LE, Bollag G, Trail PA BAY 43-9006 exhibits broad spectrum oral antitumor activity and targets the RAF/MEK/ERK pathway and receptor tyrosine kinases involved in tumor progression and angiogenesis. Cancer Res. 2004;64(19):7099–7109. 15466206 10.1158/0008-5472.CAN-04-1443

[33] Miller KD, Sweeney CJ, Sledge GW Redefining the target: chemotherapeutics as antiangiogenics. J Clin Oncol. 2001;19(4):1195–1206. 11181686 10.1200/JCO.2001.19.4.1195

[34] Golden DI, Lipson JA, Telli ML, Ford JM, Rubin DL Dynamic contrast-enhanced MRI-based biomarkers of therapeutic response in triple-negative breast cancer. J Am Med Inform Assoc. 2013;20(6):1059–1066. 23785100 10.1136/amiajnl-2012-001460 PMC3822111

[35] Ashraf A, Gaonkar B, Mies C, DeMichele A, Rosen M, Davatzikos C, Kontos D Breast DCE-MRI kinetic heterogeneity tumor markers: preliminary associations with neoadjuvant chemotherapy response. Transl Oncol. 2015;8(3):154–162. 26055172 10.1016/j.tranon.2015.03.005 PMC4487265

